# Impact of Different Occupational Noises on Static and Dynamic Postural Stability in Healthy Young Adults

**DOI:** 10.3390/ijerph22050679

**Published:** 2025-04-25

**Authors:** Kristy Gourley, Harish Chander, Asher Street Beam, Adam C. Knight

**Affiliations:** 1 Neuromechanics Laboratory, Department of Kinesiology, Mississippi State University, Starkville, MS 39762, USA; kgourley@colled.msstate.edu (K.G.); aknight@colled.msstate.edu (A.C.K.); 2 School of Health Related Professions, University of Mississippi Medical Center, Jackson, MS 39216, USA; asstreet@umc.edu

**Keywords:** occupational noises, sounds, postural stability, gait, locomotion

## Abstract

Background: Sounds that cause disturbances and perturbations to the vestibular (inner ear organ responses) and visual (acute oculomotor responses) systems can impact postural stability. The purpose of this study was to assess the impact of different types of sounds and noises on both static and dynamic PS. Methods: A total of 20 participants (12 females and 8 males; age: 21.35 ± 1.79 years; height: 170.7 ± 9.3 cm; mass: 66.725 ± 14.1 kg) were tested using the limits of stability (LOS) test on the BTrackS™ balance plate and a Timed Up and Go (TUG) test, when exposed to four different sounds and occupational noises [construction noise (CN), white noise (WN), sirens (SRs), and nature sounds (NAs)] in a randomized order with a no sounds (NSs) control performed initially (intensity range of 70–80 dB). The center of pressure (COP) total sway area (cm^2^) from the LOS and the time to completion of the TUG (seconds) were analyzed using a one-way repeated measures of analysis of variance at an alpha level of 0.05. Results: The observations demonstrated significant differences between the sounds and noises for the TUG (*p* < 0.001) but not for the LOS test (*p* = 0.406). Pairwise comparisons for the significant main effect for the TUG revealed that NSs demonstrated significantly slower time to completion compared to CN, WN, and SRs but not NAs. Conclusions: The findings suggest that the different sounds and noises did not impact static PS during the LOS test, which involved the voluntary excursion of the COP while maintaining the same base of support (BOS). However, during dynamic PS with a changing BOS while walking in the TUG, exposure to CN, SRs, and WN demonstrated a faster completion time than NSs or NAs. This finding may be attributed to the anxiety induced by the noise immersion and perception of sounds, compared to calm NAs and no sounds. The findings can aid in better understanding the impact of different occupational noises on PS and emphasize the need for better noise protection and reduction in loud work environments.

## 1. Introduction

Workplace injuries are a significant burden, costing the United States an estimated USD 167 billion in 2022 due to medical expenses, lost productivity, and other related costs [1]. Beyond the financial impact, these injuries also impose physical and emotional hardships on workers and their families. Among the leading causes of workplace injuries are slips, trips, and falls, which account for a significant portion of incidents across various industries [2,3]. High-risk occupations such as those in construction, production, protective services, and maintenance are at an especially pronounced risk. A contributing factor that potentially disrupts workers’ ability to maintain balance and postural stability (PS) is a loud work environment. As a result, there is an increased likelihood of falls in these environments [4,5,6,7,8].

The ability to maintain balance and stability while stationary or moving relies on the intricate integration of the visual, vestibular, and proprioceptive systems. The visual system allows for spatial orientation, the vestibular system detects head movements and maintains equilibrium, and the proprioceptive system monitors body positioning through sensory feedback from muscles and joints. Disruptions to one or more of these systems, whether from external factors like noise or internal impairments, can compromise balance and increase fall risk. The vestibular system, which includes the inner ear’s semicircular canals and otolith organs, is susceptible to auditory stimuli, such as noise and sound [9,10]. This has led researchers to hypothesize that auditory stimuli might influence PS, either by enhancing sensory integration or by causing distractions that impair stability [11,12,13,14]. However, studies investigating this relationship have produced conflicting findings. Some research suggests that specific sounds, such as WN, can enhance dynamic stability by stabilizing sensory inputs. At the same time, other studies report negligible or inconsistent effects, emphasizing the need for further investigation [15,16,17].

Research on the effects of sounds and noises on PS has produced mixed results, leading to investigations into the characteristics of auditory stimuli that influence these outcomes. Individual responses vary, but several studies have highlighted the role of occupational and environmental noises in affecting PS and increasing fall risk. Previous research have found that industrial workers exposed to noise levels of 82–84.99 dBA faced a 15% increase in injury risk, which rose to 34% for levels of 85–87.99 dBA [10]. Mahendra et al. (2011) observed that low-frequency noise (43–73 Hz) impaired visual acuity, coordination, balance, and spatial orientation in industrial workers [8], while Barkokébas et al. (2012) noted that construction machinery like cement mixers produced noise up to 95 dBA, exceeding these thresholds [6]. WN, on the other hand, has shown therapeutic potential for improving PS. Studies by Ross et al. (2020) and Wuehr et al. (2016) reported reduced sway and gait variability during WN exposure, with more significant improvements observed in older participants and eyes-closed conditions [18,19]. Conversely, irritating noises such as sirens, car screeches, and chainsaw sounds negatively impact PS. Błażkiewicz et al. (2024) found that these sounds increased postural irregularities during eyes-closed conditions [20], and Timofeeva et al. (2023) reported that prolonged exposure led to greater sway length, velocity, and instability [21]. Static and dynamic postural stability also tend to differ depending on the type of task being conducted, such as maintaining postural stability by staying as balanced as possible compared to intentionally moving the body to the boundaries or limits of postural stability, as well as walking in a straight direction with one’s head facing forward compared to walking with one’s head turning.

With different types of sounds having both positive and negative influences on postural stability, research on the impact of different calming sounds and noise on both static and dynamic postural stability is much warranted. The purpose of this study was to examine the effects of various types of sounds and noises on static and dynamic PS. The hypothesis was that occupational noises and other sounds would impact both static and dynamic stability.

## 2. Materials and Methods

### 2.1. Participants

A total of 20 participants (12 females and 8 males) with a mean age of 21.35 ± 1.79 years, a mean height of 170.7 ± 9.3 cm, and a mean mass of 66.725 ± 14.1 kg participated in this study. This is an experimental study with a within-subjects repeated measures design. This study’s sample size was estimated based on similar studies on this topic and methodology [11,15,16]. The study participants comprised individuals affiliated with Mississippi State University, including students and employees. All participants underwent screening to ensure they had no visual, auditory, vestibular, or proprioceptive disorders and had not experienced any lower extremity musculoskeletal injuries within the past six months. Researchers conducted the recruitment process following an IRB-approved protocol, which included the distribution of flyers and emails and word of mouth. A thorough explanation of the study details was provided to participants through an informed consent form, and their consent was obtained in compliance with the Declaration of Helsinki. This study was approved by the Institutional Review Board at Mississippi University (IRB) (Protocol # IRB 22-192 with a date of approval of 23 June 2022).

### 2.2. Instrumentation

Headphones: PowerLocus P2 (Dryanovo, Bulgaria) (loudspeaker: 40 mm; impedance: 32 ohms (Ω); loudspeaker frequency response: 110–20,000 Hz; weight: 165 g) Bluetooth noise-canceling headphones were used to administer all sound conditions to participants.

Balance Tracking Systems (BTrackS) Plate: The BTrackS plate (Balance Tracking Systems, San Diego, CA, USA), a portable force plate, was chosen for its ability to provide a variety of objective posturographic balance assessments. In this study, Assess Software version 7.5.4. was utilized, demonstrating the plate’s versatility by administering the limits of stability (LOS) test protocol. The BTrackS plate is capable of quantifying COP excursions during the LOS test.

### 2.3. Assessments

**Figure 1:**
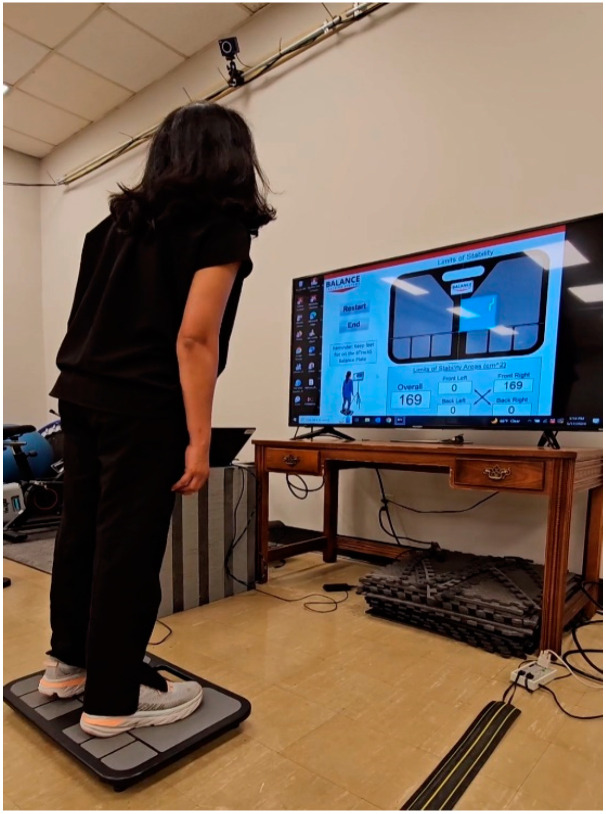
A participant performing the LOS test on the BTrackS.

Limits of Stability: During this evaluation, individuals are instructed to stand with their feet shoulder-width apart on the plate and use any means necessary to displace their COP from its resting origin point as much as possible. They were to lean as far as they believed they could without lifting their heels or the balls of their feet off the plate. Participants had exactly one minute to complete this task. Live biofeedback was available during the test to assist individuals in monitoring their movements during COP manipulation (Figure 1). The BTrackS LOS evaluation has been proven to be a reliable assessment of volitional postural control [22]. The LOS test used in this study involves a voluntary attempt at moving the COP to the boundaries or limits of postural stability to cover as much area as possible without changing the position of the feet, which is best identified as postural sway, and hence, a greater postural sway area during the LOS test implies better postural stability.

Timed Up and Go (TUG): The TUG is a widely used clinical balance assessment that involves standing up from a chair, walking three meters, turning around, walking back to the chair, and sitting down again. Many geriatric societies recommend this assessment, as it is known to be reliable [23]. It is also correlated with other clinical balance assessments and gait speed and appears to predict a person’s ability to walk independently to an outside environment with relative safety [24]. The TUG’s popularity comes mainly from its ease of use. Although it may not be as specific as other objective measures, its speed and efficiency are generally seen as acceptable [17].

### 2.4. Experimental Procedures

Upon arrival at the laboratory, the researchers instructed the participants to rest quietly while seated for five minutes before testing began. The LOS and TUG assessments were then conducted in various sound conditions. PS and locomotion assessments were carried out in the LOS test first, followed by the TUG to ensure consistent responses. To avoid potential learning effects, all participants received a practice trial on the LOS test in each sound condition. All sound conditions were administered using noise-canceling headphones to eliminate external noise pollution. The NS condition was always captured first to establish a control condition, with the remaining sounds randomized. Each sound condition lasted approximately 20 min during all assessments. Following each round of sound immersion, participants completed a sound perception questionnaire. Between each round of testing, participants had a five-minute rest period before moving on to the next sound condition.

### 2.5. Data and Statistical Analysis

COP trajectories during the LOS test from the four directional quadrants—front left, front right, back left, and back right—measured in cm^2^ were combined as the total area of the LOS (cm^2^). All data were recorded about the origin point, where an individual’s net COP remains at rest. The COP total sway area (cm^2^) from the LOS and the time to the completion of the TUG (seconds) were the dependent variables calculated and were analyzed using a one-way repeated measures of analysis of variance at an alpha level of 0.05.

## 3. Results

**Figure 2:**
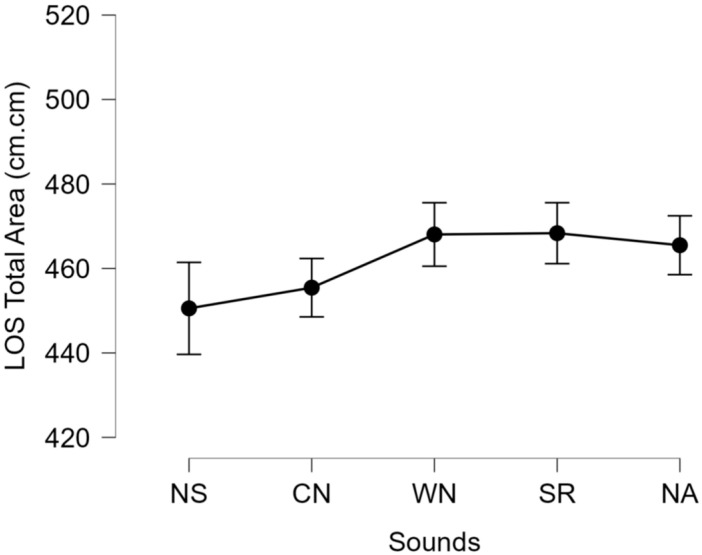
Limits of stability total area (cm^2^) from center of pressure excursions for all sounds [no sounds (NSs), construction noise (CN), white noise (WN), sirens (SRs), and nature sounds (NAs)].

**Figure 3:**
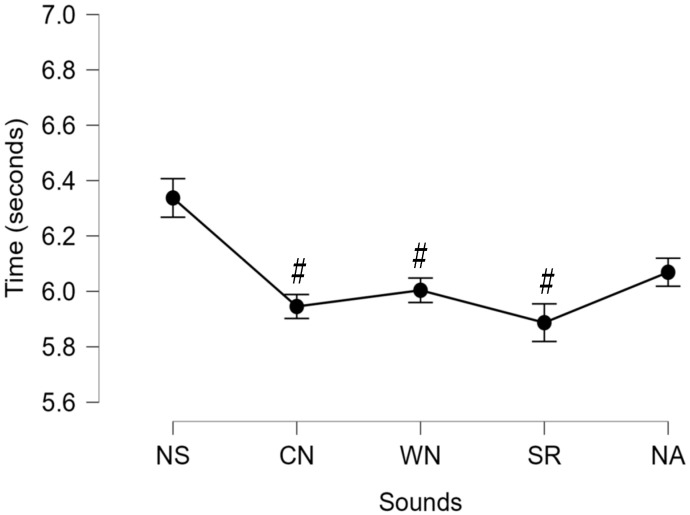
Time to completion of Timed Up and Go test (seconds) for all sounds [no sounds (NSs), construction noise (CN), white noise (WN), sirens (SRs), and nature sounds (NAs)]. # indicates significant difference at *p* < 0.05 from NSs.

Significant differences between the sounds and noises were not observed for the LOS test [F (4,76) = 1.013, *p* = 0.406; ƞ_p_^2^ = 0.051] (Figure 2). Significant differences were observed for the TUG [F (4,76) = 9.683, *p* < 0.001; ƞ_p_^2^ = 0.338] (Figure 3). Pairwise comparisons for the significant main effect for the TUG revealed that NSs demonstrated significantly slower time to completion compared to CN, WN, and SRs but not NAs.

## 4. Discussion

The objective of this study was to further investigate the impact of various noises and sounds on DS and SS. The results partially supported our hypothesis, indicating that certain auditory stimuli did affect DS. Our findings showed that exposure to CN, SRs, and WN resulted in significantly faster TUG completion times than the NA and NS conditions. This suggests improvements in DS, as slower completion times are associated with greater instability [25] or anxiety induced by specific sounds [10,11,20,26].

The results suggest that CN, WN, and SRs are associated with improved performance in dynamic tasks, as reflected by faster Timed Up and Go (TUG) completion times. Notably, the impact of CN and SRs on TUG times may be attributed to heightened cognitive arousal, enhanced processing, and improved task performance [26]. The differences in performance observed during the CN, SR, and WN TUG trials compared to the NA and NS trials may be linked to variations in autonomic arousal caused by the different emotional qualities of the auditory stimuli. Research indicates that exposure to urban sounds, such as traffic, sirens, and construction noise, increases autonomic arousal—specifically in the sympathetic nervous system—while natural sounds tend to reduce autonomic activation [10,26,27]. For instance, Laumann et al. (2003) found that subjects exposed to natural sounds, like birds and ocean waves, exhibited decreased reaction times during attention-orienting tasks compared to when they were exposed to urban sounds, such as car noise and construction [26]. Alvarsson et al. (2010) reported similar findings when their team exposed subjects to visual stimuli from urban versus natural settings [10]. If accurate, these findings could explain the faster completion times recorded during the CN, SR, and WN TUG trials and the slower TUG times noted during the NA and NS trials in this study. According to Attentional Control Theory, increased arousal under certain conditions can positively influence task engagement by allocating attentional resources to task-related processes [28]. In CN, WN, and SRs, increased arousal may have allowed participants to focus more effectively on maintaining balance during the TUG, leading to enhanced DS. However, the TUG test is commonly used in the elderly population to identify fall risk and is not necessarily used in healthy young populations. The TUG was merely chosen for this study as a standardized, simple, quick, and easy assessment of overground gait.

While increased arousal can enhance performance, this effect may be moderated by individual anxiety levels. According to Attentional Control Theory, mild arousal enhances processing efficiency, whereas excessive arousal from anxiety can reduce efficiency, leading to distraction and impaired performance. As noted by Sturnieks et al. (2016), non-anxious participants are better able to react to environmental demands, employing adaptive strategies to stabilize themselves, such as by tightening balance control or focusing attentional resources on balance tasks [29]. These adaptive responses suggest that CN, WN, and SRs may facilitate a level of arousal that enhances stability only in the absence of anxiety or distraction, allowing for appropriate attentional focus on the balance task without cognitive overload. In contrast, conditions NAs or NSs led to slower TUG times, which may be associated with a calming effect that reduced cognitive and sympathetic arousal [10,26]. The sympathetic nervous system (SNS), which modulates the activation and mobilization of resources, appears to be influenced by the type of sound present. NAs, in particular, have been shown to reduce sympathetic activity, promoting parasympathetic responses linked to relaxation and restoration [10]. Research by Laumann et al. (2003) also highlights that exposure to NAs can aid in stress recovery, potentially explaining the slower TUG times in the NA and NS conditions [26]. Reduced SNS arousal in these conditions may have decreased attentional engagement and physical mobilization, thereby reducing performance on the DS task.

For SS, our findings align with aspects of Attentional Control Theory, especially considering that this study used the LOS rather than more common quiet stance or postural sway measures for static stability assessment. Unlike the quiet stance, where minimal movement is involved and participants may not experience any immediate balance threat, the LOS task involves actively moving the COP towards the boundary of the BOS, which increases instability in subjects and likely increases attentional demand [28,30]. This movement towards the BOS perimeter can be perceived as a threat to balance, prompting participants to engage in a tightening motor response to maintain stability. This voluntary, task-focused engagement with the COP, alongside increased cognitive arousal, might contribute to heightened SS in response to auditory stimuli that subtly signal a need for greater control. In comparison, the quiet stance’s passive nature generally lacks this immediate need for stability adjustments, as minimal movement towards the perimeter of the BOS is involved. Quiet stance tasks may require fewer attentional resources than LOS tasks, making auditory stimuli less impactful.

The previous literature on the effects of the exposure of sounds on different aspects of postural stability does offer support for our current findings, with no significant difference in static postural stability [30] and no significant differences during the LOS test [31], which directly supports our current findings. The differences in postural stability can also be interpreted from a proposed “spatial hearing map theory” in which listeners build their own mental representation of the surrounding environment, which can help them to better stabilize themselves [32]. Regarding dynamic postural stability, the significant differences identified during walking in our findings is similar to improved posture with exposure to sound but only when walking with one’s head facing forward [33,34].

Overall, these findings suggest that the effects of auditory stimuli on stability may differ significantly depending on the stability task and the perceived threat to the balance level. For DS tasks like the TUG, sounds that raise arousal to an optimal level can enhance performance by engaging attentional resources. In contrast, lower arousal sounds may detract from performance by promoting relaxation over mobilization. Similarly, SS during LOS tasks may benefit from stimuli that prompt cognitive arousal, enhancing attention paid to balance control and facilitating adaptive motor responses to perceived balance threats.

### 4.1. Practical Implications

The findings from this study add to the growing body of literature on the impact of sound and noise on PS. These results and those of other studies highlight the need for better personal protective equipment for hearing and reduced loud occupational environments. Employers can help reduce workers’ risk of falls by employing better safety practices and improving noise protection. 

### 4.2. Limitations and Recommendations for Future Research

The current study has a few limitations. One limitation is this study’s narrow demographic scope, as all participants were young, college-aged individuals. Future studies should include diverse populations, such as different age groups, clinical backgrounds, and occupations, to enhance the generalizability of the findings. Additionally, the small sample size is another limitation, and increasing the sample size in future research will help to strengthen this study’s conclusions. Another limitation is the relatively short exposure time to each sound condition, which does not reflect the extended periods of occupational noise exposure experienced by workers [4,7,21]. Previous research has shown that longer noise exposure times can lead to more significant cellular changes and damage to the vestibular system [4,9]. Intermittent noise exposure also caused changes to the vestibular system, but these were less pronounced. Finally, the COP postural sway area assessed using the LOS test should not be confused with the COP postural sway area, sway velocity, or sway displacement measured during a static postural stability test, as the LOS test indicates the voluntary attempt of an individual to achieve the maximum boundaries or limits of postural stability without losing balance, and the postural sway area has a direct relationship with the postural stability assessed in an LOS test. Future research should incorporate longer exposure times to improve ecological validity and track changes in PS over time while using more complex and challenging measures of static and dynamic postural control, especially involving dual-task conditions with physical and cognitive tasks. By monitoring noise exposure duration during postural stability assessments, researchers could establish a threshold for observable changes in PS.

## 5. Conclusions

The findings suggest that the different sounds and noises did not impact static PS during the LOS, which involved the voluntary excursion of the COP while maintaining the same BOS. However, during dynamic PS with a changing BOS while walking in the TUG, exposure to CN, SRs, and WN demonstrated a faster time to completion compared to NSs or NAs. These faster completion times could be due to the anxiety caused by the noise and perception of sounds, as opposed to the calmness of NAs or silence [10,27,28]. The findings can aid in better understanding the impact of different occupational noises on PS and emphasize the need for better noise protection and reduction in loud work environments.

## Data Availability

The original contributions presented in this study are included in the article. Further inquiries can be directed to the corresponding author.
